# Supplementary material for: Closed-loop real-virtual interactions validate 3D model of social coordination in fish
Source: PLoS Comput Biol. 2026 Aug 3;22(8):e1014544. doi: 10.1371/journal.pcbi.1014544 (PMC13466055; doi:10.1371/journal.pcbi.1014544)
Supplement: Supplementary file 2 [file pcbi.1014544.s002.pdf]

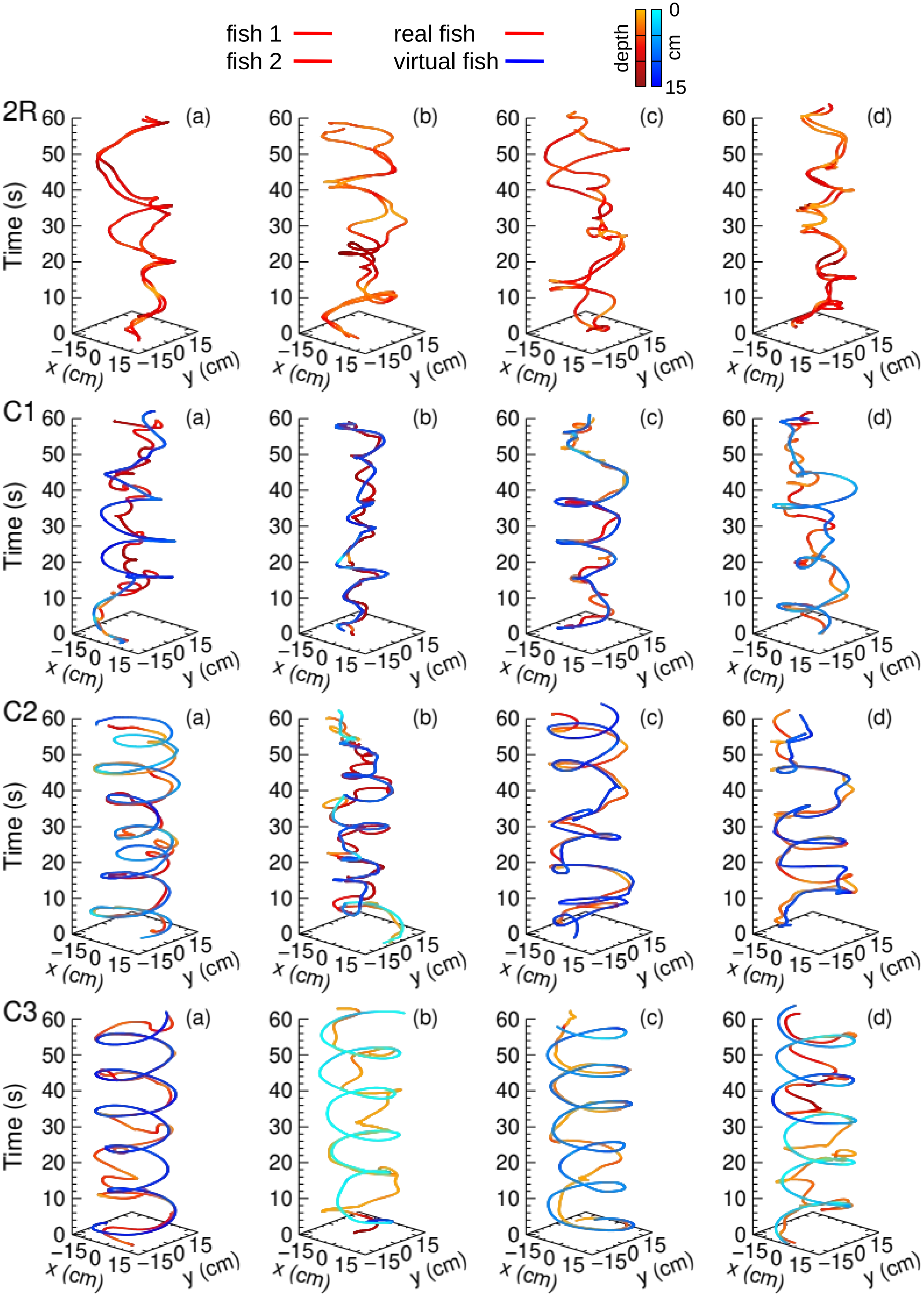
fish 1
fish 2
real fish
virtual fish
depth
cm
0
15
2R
C1
C2
C3
(a)
(b)
(c)
(d)
Time (s)
x (cm)
y (cm)
