# Supplementary material for: Closed-loop real-virtual interactions validate 3D model of social coordination in fish
Source: PLoS Comput Biol. 2026 Aug 3;22(8):e1014544. doi: 10.1371/journal.pcbi.1014544 (PMC13466055; doi:10.1371/journal.pcbi.1014544)
Supplement: Supplementary file 3 [file pcbi.1014544.s003.pdf]

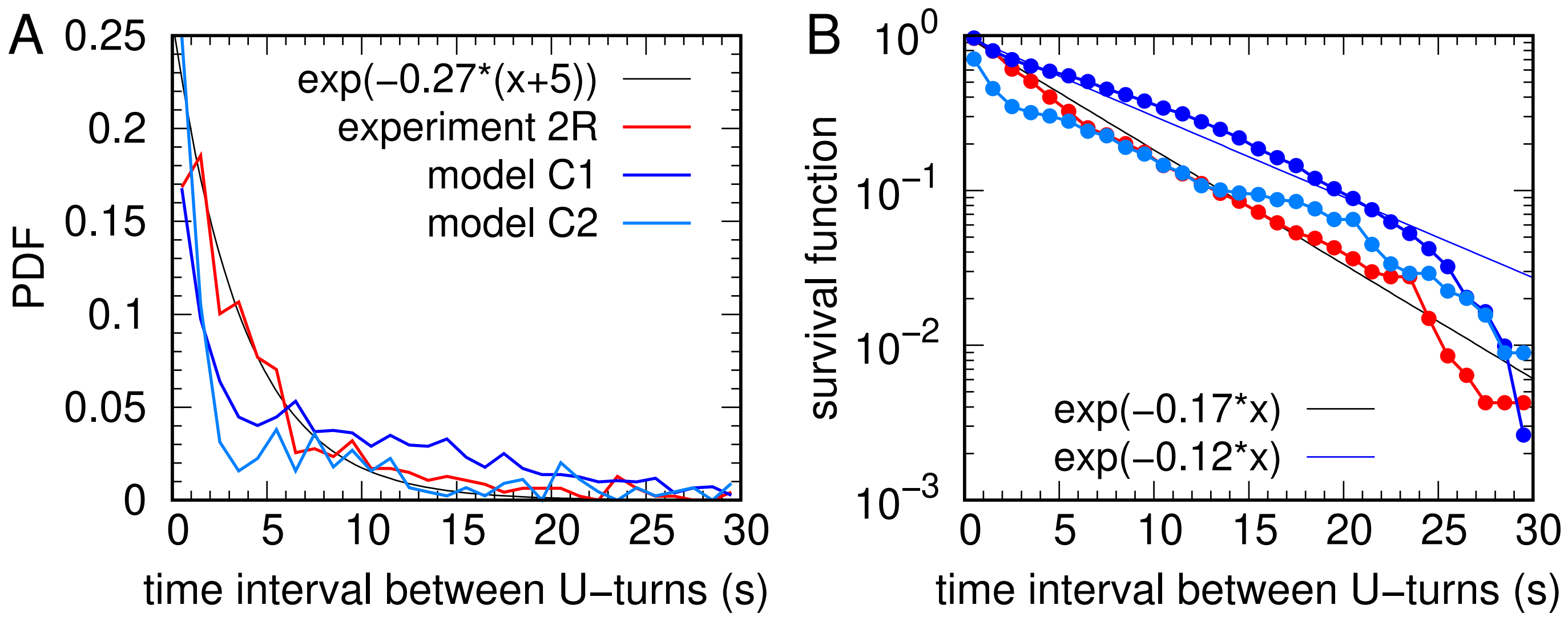

A
B
PDF
0.25
0.2
0.15
0.1
0.05
0
0
5
10
15
20
25
30
time interval between U–turns (s)
exp(−0.27*(x+5))
experiment 2R
model C1
model C2
survival function
10^0
10^{-1}
10^{-2}
10^{-3}
exp(−0.17*x)
exp(−0.12*x)
0
5
10
15
20
25
30
time interval between U–turns (s)
