# Supplementary material for: Closed-loop real-virtual interactions validate 3D model of social coordination in fish
Source: PLoS Comput Biol. 2026 Aug 3;22(8):e1014544. doi: 10.1371/journal.pcbi.1014544 (PMC13466055; doi:10.1371/journal.pcbi.1014544)
Supplement: Supplementary file 4 [file pcbi.1014544.s004.pdf]

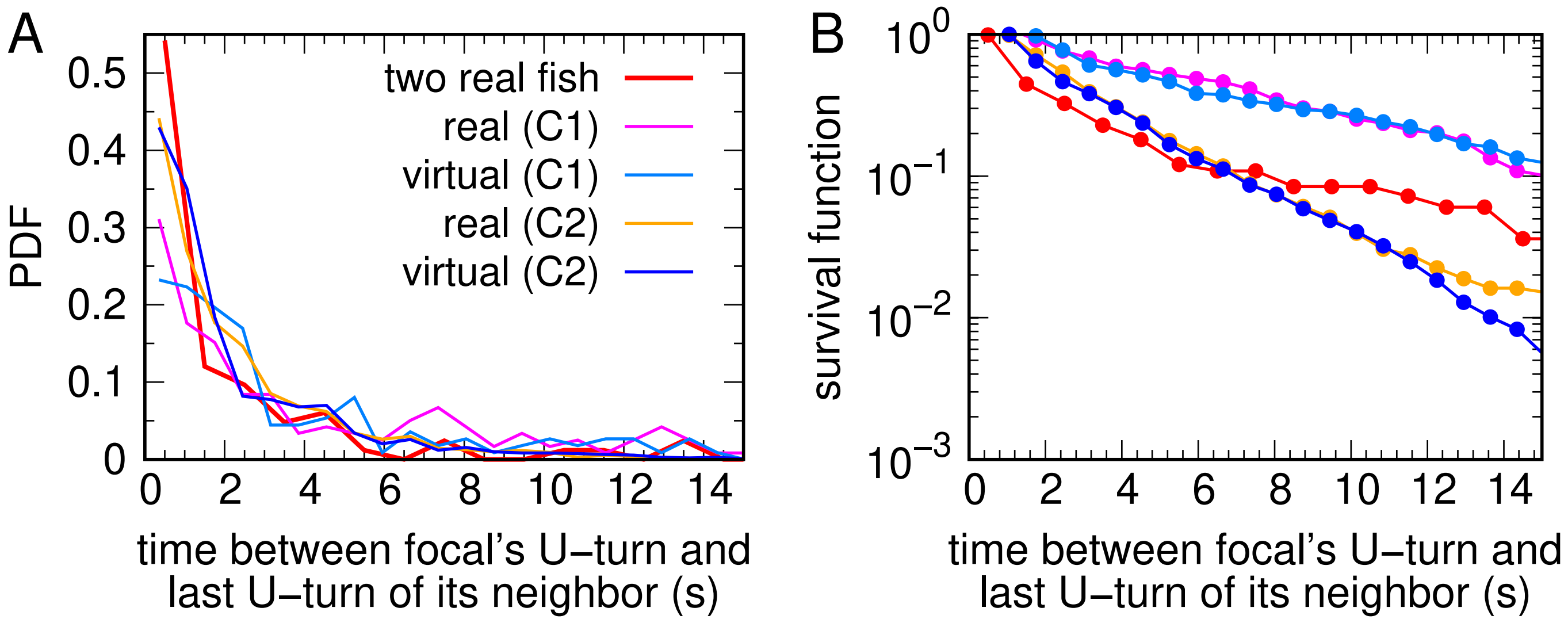
A
B
PDF
0.5
0.4
0.3
0.2
0.1
0
two real fish
real (C1)
virtual (C1)
real (C2)
virtual (C2)
0 2 4 6 8 10 12 14
time between focal's U–turn and last U–turn of its neighbor (s)
survival function
10^0
10^{-1}
10^{-2}
10^{-3}
0 2 4 6 8 10 12 14
time between focal's U–turn and last U–turn of its neighbor (s)
