# Supplementary material for: Closed-loop real-virtual interactions validate 3D model of social coordination in fish
Source: PLoS Comput Biol. 2026 Aug 3;22(8):e1014544. doi: 10.1371/journal.pcbi.1014544 (PMC13466055; doi:10.1371/journal.pcbi.1014544)
Supplement: Supplementary file 5 [file pcbi.1014544.s005.pdf]

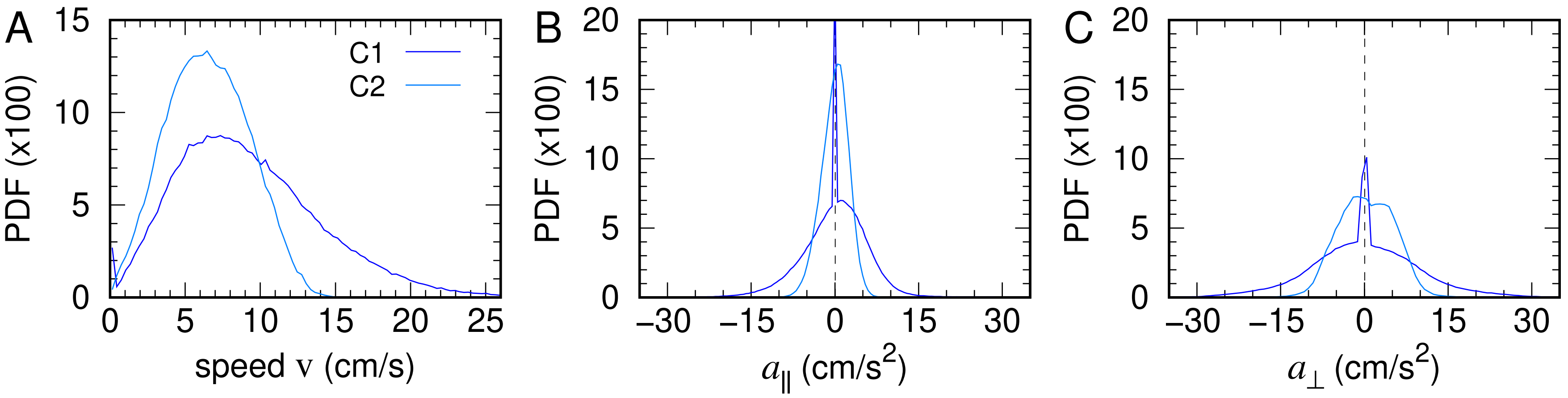

A
B
C
C1
C2
PDF (x100)
speed v (cm/s)
$a_{\parallel}$ (cm/s^2)
$a_{\perp}$ (cm/s^2)
0
5
10
15
20
25
30
−15
−30
