# Supplementary material for: Closed-loop real-virtual interactions validate 3D model of social coordination in fish
Source: PLoS Comput Biol. 2026 Aug 3;22(8):e1014544. doi: 10.1371/journal.pcbi.1014544 (PMC13466055; doi:10.1371/journal.pcbi.1014544)
Supplement: Supplementary file 6 [file pcbi.1014544.s006.pdf]

**Symbol**	**Parameter**	**Condition C1 (2 real fish)**	**C2 and C3**
$\sigma_{\parallel}$	Noise parallel component	3.4	3.3
$\sigma_{\perp}$	Noise perpendicular component	2.9	2.8
σ_z	Noise vertical component	2.9	2.35
τ_{XY}, τ_Z	Correlation times (s)	0.15	0.15
C_{v_0}	Comfort speed ($\mathrm{cm\,s^{-1}}$)	7.2	12
$D_{f_{\mathrm{Att}}}$	Attraction equilibrium distance	30	40
C_{water}	Friction modulation factor	1	0.5
C^z_{water}	Vertical friction factor	1	0.75
$C_{f_{\mathrm{w}}}$	Wall repulsion modulation factor	0.6	3.9
$C_{f_{\mathrm{Att}}}$	Attraction force factor	2	3.5
$C_{f_{\mathrm{Ali}}}$	Alignment force factor	1.4	2
$C^z_{f_{\mathrm{Att}}}$	Vertical attraction factor	1	2
