# Supplementary material for: Closed-loop real-virtual interactions validate 3D model of social coordination in fish
Source: PLoS Comput Biol. 2026 Aug 3;22(8):e1014544. doi: 10.1371/journal.pcbi.1014544 (PMC13466055; doi:10.1371/journal.pcbi.1014544)
Supplement: Supplementary file 7 [file pcbi.1014544.s007.pdf]

Supplemental Material

Closed-loop real-virtual interactions validate 3D model of social coordination in fish

Ramón Escobedo, Justine Reynaud, Renaud Bastien
Stéphane Sanchez, Clément Sire, Guy Theraulaz*

*guy.theraulaz@utoulouse.fr

Analytical expressions of the extracted social interaction functions

$$f_{\rm water}({\rm v}) = C_{\rm v}^{(1)} + C_{\rm v}^{(2)}({\rm v}-{\rm v}_0) + C_{\rm v}^{(3)}({\rm v}-{\rm v}_0)^2 + C_{\rm v}^{(4)}({\rm v}-{\rm v}_0)^3, \tag{S1}$$

$$f_{\rm adapt}({\rm v}) = C_{\rm v}^{(5)}{\rm v} + C_{\rm v}^{(6)}{\rm v}^2 + C_{\rm v}^{(7)}{\rm v}^3, \tag{S2}$$

$$f_{\rm w}(r_{\rm w}) = C_{f_{\rm w}}^{(1)} \exp\left[-\frac{r_{\rm w}}{C_{f_{\rm w}}^{(2)}} - \left(\frac{r_{\rm w}}{C_{f_{\rm w}}^{(3)}}\right)^2\right] - C_{f_{\rm w}}^{(4)}, \tag{S3}$$

$$f_{\rm rot}(r_{\rm w}) = C_{f_{\rm rot}}^{(1)} \exp\left[-\frac{r_{\rm w}}{C_{f_{\rm rot}}^{(2)}} - \left(\frac{r_{\rm w}}{C_{f_{\rm rot}}^{(3)}}\right)^2\right] - C_{f_{\rm rot}}^{(4)}, \tag{S4}$$

$$g_{\rm w}(\theta_{\rm w}) = 1 + \sum_{m=1}^{6} C_{\rm w}^{(m)} \cos(m\theta_{\rm w}), \quad g_{\rm rot}(\theta_{\rm w}) = 1 + \sum_{m=1}^{6} C_{\rm rot}^{(m)} \cos(m\theta_{\rm w}), \tag{S5}$$

$$f_{\rm Att}(d) = \frac{d - C_{f_{\rm Att}}^{(3)}}{C_{f_{\rm Att}}^{(1)}} \times \left[1 + \left(\frac{d}{C_{f_{\rm Att}}^{(2)}}\right)^2\right]^{-C_{f_{\rm Att}}^{(4)}}, \tag{S6}$$

$$\text{If } d < d_{\rm c}^{\rm Att},\ f_{\rm Att}(d) = f_{\rm Att}(d) - \left[\left(\frac{d_{\rm c}^{\rm Att}}{d}\right)^2 - 1\right] \frac{d_{\rm c}^{\rm Att}}{C_{f_{\rm Att}}^{(1)}}; \quad \text{If } f_{\rm Att} < f_{\rm Att}^{\rm min},\ f_{\rm Att} = f_{\rm Att}^{\rm min}, \tag{S7}$$

$$f_{\rm Ali}(d) = \frac{d - C_{f_{\rm Ali}}^{(3)}}{C_{f_{\rm Ali}}^{(1)}} \times \left[1 + \left(\frac{d}{C_{f_{\rm Ali}}^{(2)}}\right)^2\right]^{-C_{f_{\rm Ali}}^{(4)}}, \tag{S8}$$

$$E_{\rm Att}(\psi) = 1 + \sum_{m=1}^{6} C_{\rm Att}^{(m)} \cos(m\psi), \quad E_{\rm Ali}(\psi) = 1 + \sum_{m=1}^{6} C_{\rm Ali}^{(m)} \cos(m\psi), \tag{S9}$$

$$G_{\rm Att}(\Delta\phi) = 1 + \sum_{m=1}^{6} D_{\rm Att}^{(m)} \cos(m\Delta\phi), \quad G_{\rm Ali}(\Delta\phi) = 1 + \sum_{m=1}^{6} D_{\rm Ali}^{(m)} \cos(m\Delta\phi), \tag{S10}$$

$$f_{\rm water}^z({\rm v}_z) = C_{{\rm v}_z}^{(1)}{\rm v}_z + C_{{\rm v}_z}^{(2)}{\rm v}_z^2 + C_{{\rm v}_z}^{(3)}{\rm v}_z^3 \tag{S11}$$

$$f_z(z) = C_{f_z}^{(1)} + C_{f_z}^{(2)}(z-z_0) + C_{f_z}^{(3)}(z-z_0)^2 + C_{f_z}^{(4)}(z-z_0)^3 + C_{f_z}^{(5)}(z-z_0)^4, \tag{S12}$$

$$f_{\rm Att}^z(d_z) = C_{f_{\rm Att,z}}^{(1)} d_z + C_{f_{\rm Att,z}}^{(2)} d_z^2 + C_{f_{\rm Att,z}}^{(3)} d_z^3 + C_{f_{\rm Att,z}}^{(4)} d_z^4, \tag{S13}$$

$$f_{\rm Att,z,d}(d) = C_{f_{\rm Att,z,d}}^{(1)} \left[1 + \left(\frac{d}{C_{f_{\rm Att,z,d}}^{(2)}}\right)^{C_{f_{\rm Att,z,d}}^{(3)}}\right]^{-1}, \tag{S14}$$

$$E_{\rm Att}^z(\psi) = 1 + \sum_{m=1}^{6} C_{\rm Att,z}^{(m)} \cos(m\psi), \quad G_{\rm Att}^z(\Delta\phi) = 1 + \sum_{m=1}^{6} D_{\rm Att,z}^{(m)} \cos(m\Delta\phi). \tag{S15}$$
