# Supplementary material for: Closed-loop real-virtual interactions validate 3D model of social coordination in fish
Source: PLoS Comput Biol. 2026 Aug 3;22(8):e1014544. doi: 10.1371/journal.pcbi.1014544 (PMC13466055; doi:10.1371/journal.pcbi.1014544)
Supplement: Supplementary file 8 [file pcbi.1014544.s011.pdf]

Dear Editor,

Please find attached the revised manuscript *"Closed-loop real-virtual interactions validate 3D model of social coordination in fish"* by Ramón Escobedo, Justine Reynaud, Renaud Bastien, Stéphane Sanchez, Clément Sire, and Guy Theraulaz.

We would like to thank both reviewers for their careful reading of our manuscript and for their constructive and insightful comments. We are particularly grateful for the positive assessment of the methodological rigor and the relevance of our approach.

In this revision, we provide a "diff" PDF file highlighting the changes made in the manuscript, which are also detailed in our reply to the reviewers. Here, we summarize the most notable changes motivated by the referees' comments:

- We have revised the text to better situate our contribution within the existing literature, in particular by explicitly acknowledging prior work on social responses to virtual stimuli (e.g., Larsch and Baier, 2018) and by clarifying that our main contribution lies in the closed-loop, three-dimensional, data-driven validation of interaction rules.
- We have strengthened the description of the experimental design by introducing earlier the rationale behind the different VR conditions and by providing additional quantitative evidence of the kinematic differences between them, including new supplementary figures showing speed and acceleration distributions of the virtual fish.
- We have expanded the description of the virtual stimulus and its perceptual properties, and we now explicitly refer to previous control experiments demonstrating the absence of social responses to non-biological moving objects.
- We have refined the analysis of alignment by introducing a scalar measure of heading correlation and by clarifying the behavioral configurations underlying the observed patterns, particularly in the case of unilateral interactions;
- We have expanded the Discussion to better frame the relationship between pairwise interactions and collective three-dimensional organization, including recent complementary results on multi-neighbor interactions.

We believe these revisions significantly strengthen the manuscript by expanding the scope of the experimental results and addressing all reviewers' concerns.

Sincerely,

Ramón Escobedo et al.

Responses to Reviewers

Reviewers have raised a total of 9 points that we reproduce here in *black italicized* text. Our answers are given in blue.

Response to the comments of Referee 1

This is a rigorous and interesting paper that addresses an important question in social behavior using a technically strong and carefully executed approach. The study combines quantitative behavioral analysis, virtual-interaction experiments, and modeling in a thoughtful and compelling way, and it provides substantial new insight into the mechanisms that support coordinated behavior. Overall, I found the work to be solid, informative, and well within the scope of the journal.

A: We appreciate the Reviewer's positive assessment and constructive requests.

#1.1: *The claim regarding open-loop social responsiveness should be positioned more carefully. The general finding that fish can respond socially to non-reciprocal or open-loop visual cues is not, in itself, new. In particular, Larsch and Baier (2018) already showed in zebrafish that social affiliation can be driven by virtual visual stimuli alone, and importantly, that this can occur without reciprocity from the stimulus, since fish shoaled with projected black dots that matched fish-like motion kinetics. The novelty of the present study therefore seems to lie less in demonstrating that reciprocity is unnecessary per se, and more in the closed-loop, 3D, model-based analysis and validation of social interaction dynamics in freely swimming fish. I would encourage the authors to cite Larsch and Baier explicitly here and to calibrate their novelty claim accordingly.*

A: We fully agree with the reviewer that the ability of fish to respond socially to non-reciprocal or open-loop visual cues is not a novel finding in itself. In particular, the study by Larsch and Baier (2018) convincingly demonstrated that zebrafish can exhibit robust social affiliation toward simple visual stimuli that reproduce fish-like motion kinetics, even in the absence of reciprocal interaction. Our intention was not to claim novelty in this particular phenomenon per se, but rather to use it as a baseline against which to interpret the results obtained in our closed-loop framework. Importantly, the visual stimulus used in our study differs substantially from the minimal stimuli employed in previous work. Rather than simple moving dots, we use a realistic three-dimensional animated model of a fish that reproduces the morphology of *Hemigrammus rhodostomus*. This model is rendered in real time and projected using an anamorphic

transformation that preserves spatial coherence from the fish's perspective, creating the percept of a freely swimming conspecific within the tank. This increased level of perceptual realism is a key aspect of our approach, as it allows us to probe social interactions under conditions that more closely approximate natural sensory inputs. In particular, in section 3.1 of [23], we have shown that swapping the virtual fish for a black sphere significantly lowers the coordination of the fish motion with that of the virtual object (see also our answer to point #*1.3*).

Moreover, the central contribution of our study lies in demonstrating that a fully data-driven three-dimensional model of social interactions, inferred from real behavioral data, is capable not only of reproducing statistical properties of collective motion but also of eliciting naturalistic coordination when embedded in a real-time, closed-loop interaction with a live animal. In this sense, our work extends previous virtual reality studies by moving from stimulus-driven responses to reciprocal, dynamically coupled interactions between real and virtual agents. To clarify this point, we revised the Introduction to explicitly cite Larsch and Baier (2018) and to more carefully position our contribution.

#1.2: *The distinction between C1 and C2 should be made clearer, particularly in terms of the virtual fish's kinematics. The manuscript defines C1 as a clone with a mean speed of about 5 cm/s and C2 as a faster clone moving at about 10 cm/s, and the Methods state that C2 uses higher self-propulsion parameters. However, the actual kinematic difference between the virtual fish in C1 and C2 is not shown clearly enough for the reader to assess directly. This is important because the interpretation of the behavioral differences rests on that manipulation. I therefore think the manuscript would be strengthened by directly showing the velocity distributions of the virtual fish in C1 versus C2, and ideally also the corresponding acceleration distributions.*

A: We agree with the reviewer and have added Fig. S5 showing the PDFs of the speed and the parallel and perpendicular accelerations of the virtual fish in conditions C1 and C2, confirming the kinematic difference between conditions. We have added a short sentence at the beginning of Sec. 2.4.2.

#1.3: *The manuscript would also benefit from a stronger control experiment regarding which motion features are socially effective. Given that coordination remains substantial in C3, it would be informative to test a clearly non-biological stimulus, such as a synthetic fish moving smoothly and continuously rather than with natural burst-and-coast kinematics. This would help establish whether the observed response depends specifically on realistic motion cues.*

A: We thank the reviewer for this insightful suggestion regarding the need for a stronger control experiment to disentangle the role of biologically realistic motion features in driving social coordination. We fully agree that establishing the specificity of the observed responses to social stimuli, as opposed to generic responses to moving objects, is an important point.

In fact, such a control experiment has already been conducted within the same virtual reality framework and published in our methodological paper (in section 3.1 of [23]; Sanchez et al., 2026). In that study, we investigated the response of a real fish to a clearly non-social control stimulus consisting of a black sphere of comparable size to the tested fish, moving along a uniform circular trajectory at constant depth, speed, and distance to the edge of the tank. The results showed that fish did not follow, align with, or adjust their trajectories relative to this moving sphere, in sharp contrast to their robust following and coordination with virtual conspecifics. These findings demonstrate that the behavioral responses observed in our VR experiments are not generic reactions to moving visual objects, but are specifically triggered by socially relevant cues.

We agree that this point was not sufficiently emphasized in the present manuscript, and we revised the text accordingly.

***#1.4:** It would also help to clarify the visual properties of the projected stimulus. The manuscript refers to a "digital twin of a fish" and a "virtual conspecific," but does not clearly describe the appearance of the projected cue itself. Given that the authors also note that the extent to which fish perceive the projection as a mate may vary with context, a more explicit description of the stimulus would help the reader interpret which features may be driving the response.*

A: We thank the reviewer for this very helpful remark. We agree that the manuscript did not sufficiently describe the visual properties of the projected stimulus, and that a clearer description is important for interpreting which features drive the observed behavioral responses. We mainly refer the reader to our companion article, which provides a detailed description of the virtual reality system used in these experiments (Sanchez et al., 2026 [23]).

In our experimental setup, the projected stimulus is not a simple schematic object, but a carefully rendered three-dimensional, animated representation of a fish, designed to reproduce both the morphology and the motion characteristics of a real conspecific (shown on Figure 1A). More precisely, the virtual stimulus consists of a realistic 3D model of *Hemigrammus rhodostomus*, including body shape, coloration (notably the characteristic redhead and striped tail). This model is rendered in real time and

projected onto the inner surface of the hemispherical tank using an anamorphic transformation that continuously adapts the apparent size, orientation, and position of the stimulus to the viewpoint of the real fish. As a result, the projected image is perceived by the animal as a spatially coherent object located inside the tank rather than as a distorted image on a surface.

This point is illustrated in the VR system paper (Sanchez et al., 2026 [23]), where Fig. 5 shows (i) the real fish, (ii) the corresponding 3D animated model, and (iii) the anamorphically projected virtual fish as it appears from the fish's perspective. The projection pipeline ensures that each pixel is mapped to a physically consistent direction relative to the tracked position of the real fish, effectively reconstructing the visual scene from its point of view (see also the calibration and projection mapping procedure described in Section 2.4 of Sanchez et al., 2026).

Importantly, the system allows us to manipulate the nature of the visual stimulus. In addition to realistic fish models, we can substitute the virtual conspecific with simplified geometric objects (e.g., spheres), or modify independently the motion and morphology of the stimulus. This flexibility is key to disentangling which features (shape, motion, or interaction dynamics) are necessary to elicit social responses.

We revised the text to provide a clearer and more precise description of the projected stimulus and its perceptual properties.

Response to the comments of Referee 2

The authors investigate social interactions in fish that underlie coordinated movement and schooling behavior. They first analyze pairs of live fish swimming together to infer social interaction rules from empirical data. These inferred interactions are then implemented in a virtual fish within a virtual reality (VR) environment to test whether a real fish responds to a virtual partner in the same way as to a real conspecific.

Overall, the manuscript is well written and addresses a central question: how to infer the social interactions underlying schooling behavior. While numerous studies have examined interaction rules in flocking and schooling animals, I am not aware of previous work that combines such detailed inference with the implementation of these rules in a virtual conspecific within a VR setup.

I believe that both the approach and the results will be of interest to researchers studying collective behavior, from both experimental and theoretical perspectives, as well as to a broader community working on inferring rules of social behavior. Therefore,

the manuscript has the potential to be suitable for publication in PLOS Computational Biology. However, several issues should be addressed before it can be considered for acceptance.

A: We thank the referee for this careful and constructive reading of our manuscript, as well as for the positive assessment of the clarity, interest, and relevance of our work.

#2.1: *The authors suggest that the increase in typical swimming speed observed in the VR experiments (compared to experiments with two live fish) may be due to higher light intensity caused by the VR projection. This explanation is plausible. In retrospect, an ideal control would have been to perform the live-fish experiments under lighting conditions matching those of the VR setup. However, such matching may be technically challenging and might still not fully account for the observed speed differences, as other factors could also contribute.*

This leads to an important methodological choice. In condition C1, the virtual fish swims at 5 cm/s (the speed observed in the live-fish experiments), which results in discrepancies because the real fish swims significantly faster in the VR environment. In conditions C2 and C3, the authors increase the speed of the virtual fish to match that of the real fish in the VR setup. While this is, in principle, an elegant solution, two points deserve clarification:

First, the rationale for increasing the virtual fish's speed in C2 and C3 is introduced relatively late in the manuscript. When the three VR conditions are first described, this change appears arbitrary and may confuse the reader. I recommend briefly explaining this adjustment already at the point where the conditions are introduced.

Second, this adjustment has important implications. The improved agreement between VR and live-fish experiments after increasing the speed suggests that interaction rules inferred at lower speeds generalize, at least to some extent, to higher speeds. In other words, social interactions may be speed-independent at first order. This is a nontrivial and potentially important result that the authors should emphasize.

A: We agree that the motivation for adjusting the speed of the virtual fish in conditions C2 and C3 was not introduced early enough in the manuscript, which may indeed give the impression that this choice is arbitrary.

Our intention was to compensate for the systematic increase in swimming speed observed in the VR environment. As correctly pointed out by the reviewer, this increase is likely due, at least in part, to differences in illumination conditions induced by the

projection system. However, as the reviewer also notes, matching lighting conditions between VR and live-fish experiments is technically challenging and would not necessarily resolve the discrepancy, since other factors, such as the absence of hydrodynamic coupling or subtle perceptual differences, may also contribute.

The fact that increasing the speed of the virtual fish in conditions C2 and C3 restores quantitative agreement between VR and live-fish experiments strongly suggests that the *functional form* of the interaction rules is preserved across speeds, and that speed primarily acts as a control parameter modulating interaction strength. We agree that this is a nontrivial and important result, and we revised the manuscript to highlight this point more explicitly in both the Results and Discussion sections.

This interpretation is also consistent with our recent work (Escobedo et al., 2026) using the same experimental setup but in open-loop conditions which demonstrates that swimming speed does not fundamentally alter the *form* of social interaction rules, but rather modulates their *intensity*. Specifically, increasing speed leads to a decrease in horizontal and vertical attraction, while strengthening alignment and wall repulsion. This provides a principled justification for adjusting the speed of the virtual fish: doing so allows us to compare interaction dynamics under matched kinematic regimes, while preserving the underlying structure of the interaction rules.

We therefore revised the manuscript to introduce this rationale explicitly at the point where conditions C1–C3 are defined.

#2.2: *In Section 2.4.2 and the corresponding discussion, I found the discussion of alignment between the live fish and the virtual partner unclear. The authors appear to suggest that alignment is maximal in condition C3. This is surprising, given that alignment is only unidirectional in this condition and that the average distance between individuals is larger, which would typically weaken interactions.*

Could the reported increase in alignment instead arise from the real fish attempting to catch up from behind with the faster-moving virtual fish? A more detailed analysis of swimming trajectories and spatial configurations would help clarify which situations contribute most to the observed alignment.

More generally, I find it difficult to assess alignment based solely on distributions of relative angles. I strongly recommend introducing a scalar measure analogous to polarization used in larger groups. For pairs of fish, the average heading correlation, $\langle \cos(\Delta\varphi) \rangle$, would be appropriate. Reporting this value in the text—and possibly annotating it on the angular distribution plots—would greatly improve interpretability.

A: We thank the reviewer for this constructive comment. We agree that the interpretation of alignment in Section 2.4.2 could be clarified and that relying solely on angular distributions makes it difficult to fully assess the strength and origin of coordination. To address this point, we have extended our analysis in two complementary ways.

First, we computed a scalar measure of alignment based on the average heading correlation $\langle \cos(\Delta\varphi) \rangle$, as suggested by the reviewer. This provides a compact and intuitive quantification of alignment comparable to polarization measures used in larger groups. We find values of 0.03 for condition C1, 0.29 for C2, and 0.35 for C3, compared to 0.59 for pairs of real fish. These values have now been added to the manuscript and are reported alongside the angular distributions. This additional metric confirms that alignment remains weaker than in real pairs across all virtual conditions, but also highlights differences between conditions that were less apparent from the distributions alone.

Second, we examined in more detail the behavioral configurations contributing to alignment. As noted by the reviewer, the relatively high alignment observed in C3 might arise from the real fish attempting to catch up from behind. Our analysis supports this interpretation. In C3, the real fish spends a substantially larger fraction of time in a follower configuration (57.2%) compared to C1 (38.8%) and C2 (46.6%), as already reported in the manuscript. This indicates that the real fish is more often positioned behind the virtual partner, effectively following its trajectory. In this configuration, alignment is naturally enhanced because both individuals share a similar heading, even though the interaction is unidirectional.

To further support this interpretation, we quantified the turning dynamics of both real and virtual fish by computing U-turn rates. We find that the virtual fish in C3 performs very few U-turns (0.4 per 1000 frames) compared to C1 (1.0) and C2 (4.8), while the real fish exhibits rates of 4.5, 4.3, and 7.7 in C3, C1, and C2 respectively. This reduced turning activity makes the trajectory of the virtual fish in C3 highly predictable and easier to follow, reinforcing sustained follower behavior and thus increasing apparent alignment. Together, these results clarify that the higher alignment observed in C3 does not reflect stronger bidirectional coordination, but rather a persistent follower configuration in which the real fish tracks a predictable, non-responsive partner.

We have revised the manuscript to clarify this point and to avoid any ambiguity in the interpretation of alignment across conditions.

Minor comments

***#2.3:** Line 262ff: The authors state that "alignment is stronger when the neighbor is in front (…) and becomes anti-alignment when the neighbor is behind." If I understand correctly, anti-alignment in this context reflects the focal fish turning toward the neighbor. If so, is this truly alignment, or does it instead reflect an attraction-like interaction? Clarification would be helpful.*

A: We agree that the distinction between alignment and attraction can be subtle, and that the term "anti-alignment" may be confusing without a clearer explanation of the underlying interaction terms. In our model, attraction and alignment are explicitly separated into distinct components.

The attraction force is defined as $f_{Att}(d) \times g_{Att}(\psi) \times h_{Att}(\Delta\varphi)$ and is directed along the vector between the two fish, whereas the alignment force is defined as $f_{Ali}(d) \times g_{Ali}(\psi) \times h_{Ali}(\Delta\varphi)$ and is directed perpendicular to the speed of the focal fish (hence, only contributing to the *rotational acceleration*).

As shown in Fig. 3F, the angular modulation of attraction, $h_{Att}(\Delta\varphi)$, is essentially flat, indicating that attraction does not depend on the relative heading of the two fish. In contrast, the alignment term $h_{Ali}(\Delta\varphi)$ exhibits a pronounced peak at $\Delta\varphi = 0°$ and minima around $\Delta\varphi = \pm 160°$, which actively drives a reduction of heading differences.

The behavior described as "anti-alignment" when the neighbor is behind does not correspond to an attraction-like interaction. Rather, it reflects the fact that the alignment term produces a turning response that tends to reorient the focal fish toward the heading of the neighbor, even when their initial headings are strongly opposed. In this configuration, the effective turning direction may appear as a rotation toward the neighbor's position, but it is in fact governed by the angular dependence of the alignment interaction. The resulting motion therefore reduces the heading difference and ultimately restores alignment, rather than generating a displacement toward the neighbor as would be expected from attraction. Again, note that both interactions are directed along different vectors.

To avoid ambiguity, we have clarified this point in the manuscript by explicitly distinguishing the roles of attraction and alignment and by explaining the origin of the apparent "anti-alignment" behavior in terms of the alignment function.

***#2.4:** The authors report that fish largely match their vertical position, suggesting that interactions occur effectively in a two-dimensional plane. While interesting, observations of larger schools clearly show three-dimensional structure. This implies that vertical positioning may be regulated differently in larger groups. This point weakens the claim*

that group-level interactions can be extrapolated from pairwise interactions inferred in 3D. A brief critical discussion of this limitation would strengthen the manuscript.

A: First, note that our 3D model includes explicit interactions between fish along the vertical direction, as well as a vertical interaction of the fish with its physical environment (resulting from its interaction with the water surface and the bottom of the bowl, and possibly, also arising from a natural preference to swim at a comfort depth). Hence, we do not really agree with the implication drawn by the referee when stating that "*fish largely match their vertical position, **suggesting that interactions occur effectively in a two-dimensional plane**".* It is actually these *vertical interactions* that produce a smaller extension of the pairs of real fish or virtual-real fish pairs along the vertical direction than along the horizontal direction. Also note that large schools in unbounded space are usually non-isotropic, often extending much more widely in the horizontal XY directions than along the vertical direction (the "bait ball" structure observed in some species is an obvious exception). Moreover, a fish school in "unbounded" XY space and bounded space in the Z direction (by the water depth, biological constraints and/or a preferential depth of the considered species), the average vertical velocity over a sufficiently long period is necessarily null despite some schools being able to travel thousands of kilometers in the XY direction (in other words, the mean fish heading is along the horizontal direction). Hence, there is indeed an intrinsic asymmetry between the XY and Z directions, even in natural settings. Finally, the present study focuses on a specific species, the red nose tetra, and other species may interact via more isotropic interactions.

Yet, even for the considered species, and due to the finite range of the vertical (and horizontal interactions), it is expected that, as the group size increases, its spatial extension should also increase in the horizontal *and* vertical directions. A natural next step is therefore to understand how interactions combine when multiple neighbors are present. We have precisely addressed this question in a complementary study using the same closed-loop virtual reality framework (Kang et al., *Decoding social integration in schooling fish using closed-loop real–virtual interactions*), currently under review and available as a preprint on bioRxiv:

https://doi.org/10.64898/2026.03.12.711426).

In that work, we show that real fish behave as if they respond primarily to a single most influential neighbor, whose identity dynamically changes over time. Remarkably, this selective interaction strategy is sufficient to sustain coordinated group motion. In addition, we find that these interactions lead to a three-dimensional spatial organization, with vertical separations between fish extending up to approximately 8 cm in groups of 5 fish.

These results provide a clear interpretation of the apparent discrepancy raised by the reviewer. Pairwise interactions reveal a tendency toward vertical alignment, but in larger groups, the dynamic selection of influential neighbors introduces variability and flexibility in depth positioning, ultimately giving rise to a genuinely three-dimensional structure. Rather than weakening our approach, this highlights the importance of combining pairwise characterization with multi-agent integration to fully understand collective behavior.

We revised the Discussion to clarify the scope of our conclusions and to place our results in the broader context of multi-individual interactions.

#2.5: *Based on comparisons of conditions C1–C3, the authors conclude that speed is a crucial determinant of social interactions underlying schooling. While the VR-based results are novel, previous studies have also highlighted the importance of speed (some of which are already cited). In addition, the authors may want to consider including the following recent and relevant study: Puy et al., PNAS 121 (2024).*

A: We thank the reviewer for this remark and for pointing out the recent study by Puy et al. (2024). We fully agree that the role of speed in shaping social interactions has been well documented, including in studies already cited in the manuscript.

Our intention was not to present speed as a novel factor per se, but to demonstrate its role within a closed-loop experimental framework, where its effect can be tested causally. In this context, our results show that increasing the speed of the virtual partner enhances coordination and alignment, in agreement with previous findings, including the speed-induced leadership and selective interactions reported by Puy et al. (2024).

Importantly, our results further indicate that speed does not alter the *functional form* of social interactions, but rather modulates their *intensity*. This interpretation is supported by our recent open-loop VR study (Escobedo et al., 2026), which shows that increasing speed weakens attraction while strengthening alignment and wall repulsion. Together, these findings suggest that speed acts as a control parameter that reweights interaction components without changing their underlying structure.

We revised the manuscript accordingly by explicitly citing Puy et al. (2024) and clarifying that our contribution lies in providing a causal, closed-loop validation of how speed modulates social interaction strength.

Cited references

Escobedo, R., Reynaud, J., Sanchez, S., Sire, C. & Theraulaz, G. 2026. Swimming speed of schooling fish controls social interaction strength in closed-loop immersive virtual reality. *Proceedings of the Royal Society of London Series B-Biological Sciences* (in press).

Kang, Z., Escobedo, R., Combe, M., Sanchez, S., Sire, C. & Theraulaz, G. Decoding social integration in schooling fish using closed-loop real–virtual interactions. *BioRxiv.* 2026. doi:10.64898/2026.03.12.711426.
