# Supplementary material for: Closed-loop real-virtual interactions validate 3D model of social coordination in fish
Source: PLoS Comput Biol. 2026 Aug 3;22(8):e1014544. doi: 10.1371/journal.pcbi.1014544 (PMC13466055; doi:10.1371/journal.pcbi.1014544)
Supplement: Supplementary file 9 [file pcbi.1014544.s012.pdf]

Dear Editor,

Please find attached the revised version of our manuscript entitled *"Closed-loop real-virtual interactions validate a 3D model of social coordination in fish"*.

We have carefully addressed all the points raised by Referee 2 in the attached rebuttal letter and provided detailed responses to his concerns. We have also revised the statement in line 248, as requested by the reviewer.

We hope that the revised manuscript and our responses satisfactorily address the remaining concerns and that the manuscript is now suitable for publication in *Plos Computational Biology*.

Sincerely

Ramón Escobedo et al.

Responses to Reviewers

Reviewer 2 has raised one remaining point that we reproduce below in *black italicized* text. Our answer is given in blue.

Response to the comments of Referee 1

The authors have addressed all of my comments in a satisfactory manner. I congratulate them on a thoughtful and well-executed study.

A: We thank the referee for the careful evaluation of our manuscript and for the positive assessment of our work. We are pleased that our revisions have satisfactorily addressed all comments and concerns. We appreciate the referee's constructive feedback, which helped improve the clarity and quality of the manuscript.

Response to the comments of Referee 2

While the authors addressed most of my comments to my satisfaction, I am not satisfied with their response regarding the explanation of anti-alignment as opposed to attraction.

The authors state in their response that, in their model, the attraction force is a vector acting along the connecting vector, while the alignment force acts perpendicular to the speed of the focal fish, contributing only to rotational acceleration. This in itself does not rule out the possibility that the observed anti-alignment results from some sort of "additional" attraction response conditioned on the particular spatial configuration. To make my point clear: if we consider a self-propelled agent with constant speed, then any vectorial force will, by definition, only be able to induce rotations (i.e., act perpendicular to the velocity vector).

For variable speed, an effective vectorial force can act on both rotation and speed modulation. There is no a priori reason that a resulting social interaction must act in the same way as a physical force on a self-propelled body — that is, that the magnitude of the change in angle and speed is directly given by the projection of the total force onto the two perpendicular unit vectors (velocity unit vector e_para and angle unit vector e_perp). In general, it is possible that an attractive interaction may act differently on speed |v| than on the angle phi. I clarify my point by rewriting the equation of motion in polar coordinates in 2D for a total force F:

(1) d|v|/dt = alpha_v * < F . e_para>
(2) |v| * dphi/dt = alpha_phi * **< F . e_perp>** (**equation corrected by the authors)**

where < . > denotes the scalar product of two vectors.

For a physical force, alpha_v = alpha_phi = 1, but in the general case of a social interaction, these two factors need not be the same. To make things even more complicated, the alphas could depend on relative position angles or even relative orientations. The authors themselves use this approach — for example, in their modeling of the wall force, which they include in Eq. 1 with two separate force terms, one for rotation only (equivalent to setting alpha_v = 0).

Figure 3E clearly shows a negative g_align for |psi| > 120°, with h_ali > 0 for all Delta phi. This indicates that the overall term without sin(Delta phi) is negative. Instead of reducing the heading difference for this particular configuration, it increases Delta phi, which contradicts the statement added in line 248 that it "tends to reduce heading differences." I still do not see how, even with the additional assumption about relative heading difference, this apparent anti-alignment could not be some extra attraction-like response for these particular relative position configurations.

Of course, this is not a simple attractive interaction, as it only acts when the neighbor is behind the focal fish. However, interestingly, the sign change of the alignment interaction in Fig. 3 occurs at psi values where the inferred attraction function has a maximum, and also h_att > h_ali (Fig. 3F). Thus, the apparent slight decrease in g_att for neighbors being behind (|psi| > 120°) might occur because the "anti-alignment" takes over some of the "job" of turning the focal fish toward the neighbor. In the end, what matters is the behavioral response, and it appears to be non-trivially complex. I am now starting to wonder whether the model formulation used in the paper — based on symmetries and empirical observation of anisotropic response, which at first glance makes perfect sense to me — might also constrain inference in a particular way, and whether there could be some alternative general formulation of the interactions that would constrain it differently, thus leading to a potentially different interpretation of the observed anti-alignment pattern.

In the end, I would expect the authors to reconsider their interpretation of anti-alignment, either by providing an actual argument as to why it cannot be an attraction-like response, or providing a short critical discussion on the potential unclear origin, and to correct the imprecise statement in line 248 that it "tends to reduce heading differences."

A: Mathematically, in a particle model, an attraction force is directed **from a focal individual 1 toward its attracting neighbor 2 (understanding that the point particles are located at the center of mass of the actual objects)**. In addition, **a rotational force is, again by definition, purely directed along the normal to its velocity**. A typical example of an attractive force in physics is the gravitational force between two masses or the electrostatic force between two opposite-charge particles. Corresponding to the latter case, the force exerted by a magnetic field on a charged particle is a rotational force purely directed along the normal to the velocity (and also normal to the magnetic field). A similar but simpler situation arises when the particle moves in a plane and is submitted to a magnetic field perpendicular to the plane.

The referee is correct that an attractive force has a component along the direction of the velocity and also along the normal to the velocity, but these two components **are not independent, being the scalar product of the *same force* with e_para and e_perp**.

The referee argues that for social forces, alpha_v and alpha_phi may not be equal to 1 or may not be even the same. This totally negates the definition of an attractive force, which, by definition, is directed from a particle to another, and hence should act as such in the corresponding equation of motion. Attraction is, by construction, the component that changes the inter-individual distance and is hence directed along the vector between 1 and 2. Any additional turning induced by this same interaction is simply the geometrical projection of this radial force onto the direction normal to the focal velocity and does not constitute an independent behavioral interaction.

Introducing different alphas amounts to an effective attractive force, which is not anymore directed along the vector between 1 and 2. Note, however, that even if one authorizes different alphas, the effect of the original force can still be interpreted as the sum of a true attractive force (i.e., directed along the vector between 1 and 2) and a purely rotational force (see the corrected Eqs. (1-2) above):

(3) *d|v|/dt = alpha_v * < F . e_para>*

(4) *|v| * dphi/dt = alpha_v * < F . e_perp> + (alpha_phi - alpha_v) * < F . e_perp>*

With this trivial reformulation, one recovers a standard equation with an additional purely rotational force proportional to *(alpha_phi - alpha_v)*. But the formulation of the referee does not make real sense, because a force can only be defined (and measured experimentally) through its impact on the acceleration. In other words, the formulation of the referee involves a force F supposedly defined independently from the equation of motion and one defined from its impact on the acceleration depending on some hypothetical alphas. The referee should realize that there is no way to measure the alphas, and that one can only access, for instance, to f_para = *alpha_v * < F . e_para>*,

from its impact on the acceleration, which is ultimately the relevant effective force along the velocity direction (and how a force is actually defined).

At the end of the day, whatever the origin of the (social or physical) forces between two particles in the plane, one can always express it as having a component along the vector between 1 and 2 plus a component along the normal to the velocity of 1 (the 2 vectors form a basis of the plane, except in the extremely rare pathological case when both are exactly aligned). In practice, our reconstruction procedure measures these two components. The first one is interpreted as an attractive/repulsive force, and the second as a rotational force.

The referee mentions that *the alphas could depend on relative position angles or even relative orientations*, but should realize that this variation can be simply absorbed in the attraction and rotation interactions, which indeed do depend on all these quantities in our approach.

In conclusion, the existence (or not) of underlying coefficients such as *alpha_v* and *alpha_phi* is ultimately a moot point, because only the resulting acceleration can be measured experimentally. Our reconstruction procedure infers the interaction terms directly from the equation of motion, i.e., from their contribution to the observed acceleration. Any underlying formulation involving different alphas can always be recast into an equivalent and unique decomposition in terms of a true attractive force (i.e., directed between individuals 1 and 2) and a pure rotational force (perpendicular to the velocity of 1), yielding exactly the same measurable dynamics. Consequently, the decomposition used in this work is not an arbitrary interpretation but a unique decomposition/representation of the experimentally identifiable interaction terms and their impact on the fish's motion (i.e., their acceleration).

Finally as requested by the reviewer, we have revised the statement in line 248,
