# Supplementary material for: Closed-loop real-virtual interactions validate 3D model of social coordination in fish
Source: PLoS Comput Biol. 2026 Aug 3;22(8):e1014544. doi: 10.1371/journal.pcbi.1014544 (PMC13466055; doi:10.1371/journal.pcbi.1014544)
Supplement: Peer review history

# Peer review: Decision Letter 0

**DOI:** 10.1371/journal.pcbi.1014544.r001

**Authors:** Natalia L. Komarova, Nir Gov

24 Apr 2026

Closed-loop real-virtual interactions validate 3D model of social coordination in fish

PLOS Computational Biology

Dear Dr. Theraulaz,

Thank you for submitting your manuscript to PLOS Computational Biology. After careful consideration, we feel that it has merit but does not fully meet PLOS Computational Biology's publication criteria as it currently stands. Therefore, we invite you to submit a revised version of the manuscript that addresses the points raised during the review process.

Please submit your revised manuscript by Jun 24 2026 11:59PM. If you will need more time than this to complete your revisions, please reply to this message or contact the journal office at ploscompbiol@plos.org. When you're ready to submit your revision, log on to https://www.editorialmanager.com/pcompbiol/ and select the 'Submissions Needing Revision' folder to locate your manuscript file.

Please include the following items when submitting your revised manuscript:

* A letter that responds to each point raised by the editor and reviewer(s). You should upload this letter as a separate file labeled 'Response to Reviewers'. This file does not need to include responses to formatting updates and technical items listed in the 'Journal Requirements' section below.

* A marked-up copy of your manuscript that highlights changes made to the original version. You should upload this as a separate file labeled 'Revised Manuscript with Track Changes'.

* An unmarked version of your revised paper without tracked changes. You should upload this as a separate file labeled 'Manuscript'.

If you would like to make changes to your financial disclosure, competing interests statement, or data availability statement, please make these updates within the submission form at the time of resubmission. Guidelines for resubmitting your figure files are available below the reviewer comments at the end of this letter

We look forward to receiving your revised manuscript.

Kind regards,

Nir Gov

Academic Editor

PLOS Computational Biology

Natalia Komarova

Section Editor

PLOS Computational Biology

**Journal Requirements:**

1) We ask that a manuscript source file is provided at Revision. Please upload your manuscript file as a .doc, .docx, .rtf or .tex. If you are providing a .tex file, please upload it under the item type u2018LaTeX Source Fileu2019 and leave your .pdf version as the item type u2018Manuscriptu2019.

2) Please provide an Author Summary. This should appear in your manuscript between the Abstract (if applicable) and the Introduction, and should be 150-200 words long. The aim should be to make your findings accessible to a wide audience that includes both scientists and non-scientists. Sample summaries can be found on our website under Submission Guidelines:

https://journals.plos.org/ploscompbiol/s/submission-guidelines#loc-parts-of-a-submission

3) Please upload all main figures as separate Figure files in .tif or .eps format. For more information about how to convert and format your figure files please see our guidelines:

https://journals.plos.org/ploscompbiol/s/figures

4) Please amend your detailed Financial Disclosure statement. This is published with the article. It must therefore be completed in full sentences and contain the exact wording you wish to be published.

1) State the initials, alongside each funding source, of each author to receive each grant. For example: "This work was supported by the National Institutes of Health (####### to AM; ###### to CJ) and the National Science Foundation (###### to AM)."

2) State what role the funders took in the study. If the funders had no role in your study, please state: "The funders had no role in study design, data collection and analysis, decision to publish, or preparation of the manuscript.".

If you did not receive any funding for this study, please simply state: u201cThe authors received no specific funding for this work.u201d

5)  Figures 1 and Striking Image: Please confirm (a) that you are the photographer; or (b) provide written permission from the photographer to publish the photo(s) under our CC BY 4.0 license.

**Reviewers' comments:**

Reviewer's Responses to Questions

**Comments to the Authors:**

**Please note here if the review is uploaded as an attachment.**

Reviewer #1: This is a rigorous and interesting paper that addresses an important question in social behavior using a technically strong and carefully executed approach. The study combines quantitative behavioral analysis, virtual-interaction experiments, and modeling in a thoughtful and compelling way, and it provides substantial new insight into the mechanisms that support coordinated behavior. Overall, I found the work to be solid, informative, and well within the scope of the journal.

1. The claim regarding open-loop social responsiveness should be positioned more carefully. The general finding that fish can respond socially to non-reciprocal or open-loop visual cues is not, in itself, new. In particular, Larsch and Baier (2018) already showed in zebrafish that social affiliation can be driven by virtual visual stimuli alone, and importantly, that this can occur without reciprocity from the stimulus, since fish shoaled with projected black dots that matched fish-like motion kinetics. The novelty of the present study therefore seems to lie less in demonstrating that reciprocity is unnecessary per se, and more in the closed-loop, 3D, model-based analysis and validation of social interaction dynamics in freely swimming fish. I would encourage the authors to cite Larsch and Baier explicitly here and to calibrate their novelty claim accordingly.

2. The distinction between C1 and C2 should be made clearer, particularly in terms of the virtual fish’s kinematics. The manuscript defines C1 as a clone with a mean speed of about 5 cm/s and C2 as a faster clone moving at about 10 cm/s, and the Methods state that C2 uses higher self-propulsion parameters. However, the actual kinematic difference between the virtual fish in C1 and C2 is not shown clearly enough for the reader to assess directly. This is important because the interpretation of the behavioral differences rests on that manipulation. I therefore think the manuscript would be strengthened by directly showing the velocity distributions of the virtual fish in C1 versus C2, and ideally also the corresponding acceleration distributions.

3. The manuscript would also benefit from a stronger control experiment regarding which motion features are socially effective. Given that coordination remains substantial in C3, it would be informative to test a clearly non-biological stimulus, such as a synthetic fish moving smoothly and continuously rather than with natural burst-and-coast kinematics. This would help establish whether the observed response depends specifically on realistic motion cues.

4. It would also help to clarify the visual properties of the projected stimulus. The manuscript refers to a “digital twin of a fish” and a “virtual conspecific,” but does not clearly describe the appearance of the projected cue itself. Given that the authors also note that the extent to which fish perceive the projection as a mate may vary with context, a more explicit description of the stimulus would help the reader interpret which features may be driving the response.

Reviewer #2: The authors investigate social interactions in fish that underlie coordinated movement and schooling behavior. They first analyze pairs of live fish swimming together to infer social interaction rules from empirical data. These inferred interactions are then implemented in a virtual fish within a virtual reality (VR) environment to test whether a real fish responds to a virtual partner in the same way as to a real conspecific.

Overall, the manuscript is well written and addresses a central question: how to infer the social interactions underlying schooling behavior. While numerous studies have examined interaction rules in flocking and schooling animals, I am not aware of previous work that combines such detailed inference with the implementation of these rules in a virtual conspecific within a VR setup.

I believe that both the approach and the results will be of interest to researchers studying collective behavior, from both experimental and theoretical perspectives, as well as to a broader community working on inferring rules of social behavior. Therefore, the manuscript has the potential to be suitable for publication in PLOS Computational Biology. However, several issues should be addressed before it can be considered for acceptance.

Major comments

1) The authors suggest that the increase in typical swimming speed observed in the VR experiments (compared to experiments with two live fish) may be due to higher light intensity caused by the VR projection. This explanation is plausible. In retrospect, an ideal control would have been to perform the live-fish experiments under lighting conditions matching those of the VR setup. However, such matching may be technically challenging and might still not fully account for the observed speed differences, as other factors could also contribute.

This leads to an important methodological choice. In condition C1, the virtual fish swims at 5 cm/s (the speed observed in the live-fish experiments), which results in discrepancies because the real fish swims significantly faster in the VR environment. In conditions C2 and C3, the authors increase the speed of the virtual fish to match that of the real fish in the VR setup. While this is, in principle, an elegant solution, two points deserve clarification:

First, the rationale for increasing the virtual fish’s speed in C2 and C3 is introduced relatively late in the manuscript. When the three VR conditions are first described, this change appears arbitrary and may confuse the reader. I recommend briefly explaining this adjustment already at the point where the conditions are introduced.

Second, this adjustment has important implications. The improved agreement between VR and live-fish experiments after increasing the speed suggests that interaction rules inferred at lower speeds generalize, at least to some extent, to higher speeds. In other words, social interactions may be speed-independent at first order. This is a nontrivial and potentially important result that the authors should emphasize.

2) In Section 2.4.2 and the corresponding discussion, I found the discussion of alignment between the live fish and the virtual partner unclear. The authors appear to suggest that alignment is maximal in condition C3. This is surprising, given that alignment is only unidirectional in this condition and that the average distance between individuals is larger, which would typically weaken interactions.

Could the reported increase in alignment instead arise from the real fish attempting to catch up from behind with the faster-moving virtual fish? A more detailed analysis of swimming trajectories and spatial configurations would help clarify which situations contribute most to the observed alignment.

More generally, I find it difficult to assess alignment based solely on distributions of relative angles. I strongly recommend introducing a scalar measure analogous to polarization used in larger groups. For pairs of fish, the average heading correlation, 〈cos(Δϕ)〉, would be appropriate. Reporting this value in the text—and possibly annotating it on the angular distribution plots—would greatly improve interpretability.

Minor comments

Line 262ff: The authors state that “alignment is stronger when the neighbor is in front (…) and becomes anti-alignment when the neighbor is behind.” If I understand correctly, anti-alignment in this context reflects the focal fish turning toward the neighbor. If so, is this truly alignment, or does it instead reflect an attraction-like interaction? Clarification would be helpful.

The authors report that fish largely match their vertical position, suggesting that interactions occur effectively in a two-dimensional plane. While interesting, observations of larger schools clearly show three-dimensional structure. This implies that vertical positioning may be regulated differently in larger groups. This point weakens the claim that group-level interactions can be extrapolated from pairwise interactions inferred in 3D. A brief critical discussion of this limitation would strengthen the manuscript.

Based on comparisons of conditions C1–C3, the authors conclude that speed is a crucial determinant of social interactions underlying schooling. While the VR-based results are novel, previous studies have also highlighted the importance of speed (some of which are already cited). In addition, the authors may want to consider including the following recent and relevant study:

Puy et al., PNAS 121 (2024)

**********

**Have the authors made all data and (if applicable) computational code underlying the findings in their manuscript fully available?**

The PLOS Data policy requires authors to make all data and code underlying the findings described in their manuscript fully available without restriction, with rare exception (please refer to the Data Availability Statement in the manuscript PDF file). The data and code should be provided as part of the manuscript or its supporting information, or deposited to a public repository. For example, in addition to summary statistics, the data points behind means, medians and variance measures should be available. If there are restrictions on publicly sharing data or code —e.g. participant privacy or use of data from a third party—those must be specified.

Reviewer #1: Yes

Reviewer #2: **No:**  I did not find any information on availability of the computational code, neither for the social interaction inferrence or the simulation model.

**********

PLOS authors have the option to publish the peer review history of their article (what does this mean?). If published, this will include your full peer review and any attached files.

If you choose “no”, your identity will remain anonymous but your review may still be made public.

**Do you want your identity to be public for this peer review?**  For information about this choice, including consent withdrawal, please see our Privacy Policy.

Reviewer #1: No

Reviewer #2: No

[NOTE: If reviewer comments were submitted as an attachment file, they will be attached to this email and accessible via the submission site. Please log into your account, locate the manuscript record, and check for the action link "View Attachments". If this link does not appear, there are no attachment files.]

**Figure resubmission:**

**Reproducibility:**

?>

# Author comment: Author response to Decision Letter 1

**DOI:** 10.1371/journal.pcbi.1014544.r002

13 May 2026

Attachment Submitted filename: Response to Reviewers_R1.pdf

# Peer review: Decision Letter 1

**DOI:** 10.1371/journal.pcbi.1014544.r003

**Authors:** Natalia L. Komarova, Nir Gov

18 Jun 2026

PCOMPBIOL-D-26-00551R1

Closed-loop real-virtual interactions validate 3D model of social coordination in fish

PLOS Computational Biology

Dear Dr. Theraulaz,

Thank you for submitting your manuscript to PLOS Computational Biology. After careful consideration, we feel that it has merit but does not fully meet PLOS Computational Biology's publication criteria as it currently stands. Therefore, we invite you to submit a revised version of the manuscript that addresses the points raised during the review process.

Please submit your revised manuscript by Aug 18 2026 11:59PM. If you will need more time than this to complete your revisions, please reply to this message or contact the journal office at ploscompbiol@plos.org. When you're ready to submit your revision, log on to https://www.editorialmanager.com/pcompbiol/ and select the 'Submissions Needing Revision' folder to locate your manuscript file.

Please include the following items when submitting your revised manuscript:

* A letter that responds to each point raised by the editor and reviewer(s). You should upload this letter as a separate file labeled 'Response to Reviewers'. This file does not need to include responses to formatting updates and technical items listed in the 'Journal Requirements' section below.

* A marked-up copy of your manuscript that highlights changes made to the original version. You should upload this as a separate file labeled 'Revised Manuscript with Track Changes'.

* An unmarked version of your revised paper without tracked changes. You should upload this as a separate file labeled 'Manuscript'.

If you would like to make changes to your financial disclosure, competing interests statement, or data availability statement, please make these updates within the submission form at the time of resubmission. Guidelines for resubmitting your figure files are available below the reviewer comments at the end of this letter.

As the corresponding author, your ORCID iD is verified in the submission system and will appear in the published article. PLOS supports the use of ORCID, and we encourage all coauthors to register for an ORCID iD and use it as well. Please encourage your coauthors to verify their ORCID iD within the submission system before final acceptance, as unverified ORCID iDs will not appear in the published article. Only the individual author can complete the verification step; PLOS staff cannot verify ORCID iDs on behalf of authors.

We look forward to receiving your revised manuscript.

Kind regards,

Nir Gov

Academic Editor

PLOS Computational Biology

Natalia Komarova

Section Editor

PLOS Computational Biology

**Additional Editor Comments (if provided):**

**Journal Requirements:**

If the reviewer comments include a recommendation to cite specific previously published works, please review and evaluate these publications to determine whether they are relevant and should be cited. There is no requirement to cite these works unless the editor has indicated otherwise.

1) Please amend your detailed Financial Disclosure statement. This is published with the article. It must therefore be completed in full sentences and contain the exact wording you wish to be published.

i) State the initials, alongside each funding source, of each author to receive each grant. For example: "This work was supported by the National Institutes of Health (####### to AM; ###### to CJ) and the National Science Foundation (###### to AM).".

If you did not receive any funding for this study, please simply state: u201cThe authors received no specific funding for this work.u201d

**Reviewers' comments:**

Reviewer's Responses to Questions

**Comments to the Authors:**

**Please note here if the review is uploaded as an attachment.**

Reviewer #1: The authors have addressed all of my comments in a satisfactory manner. I congratulate them on a thoughtful and well-executed study.

Reviewer #2: While the authors addressed most of my comments to my satisfaction, I am not satisfied with their response regarding the explanation of anti-alignment as opposed to attraction.

The authors state in their response that, in their model, the attraction force is a vector acting along the connecting vector, while the alignment force acts perpendicular to the speed of the focal fish, contributing only to rotational acceleration. This in itself does not rule out the possibility that the observed anti-alignment results from some sort of "additional" attraction response conditioned on the particular spatial configuration. To make my point clear: if we consider a self-propelled agent with constant speed, then any vectorial force will, by definition, only be able to induce rotations (i.e., act perpendicular to the velocity vector).

For variable speed, an effective vectorial force can act on both rotation and speed modulation. There is no a priori reason that a resulting social interaction must act in the same way as a physical force on a self-propelled body — that is, that the magnitude of the change in angle and speed is directly given by the projection of the total force onto the two perpendicular unit vectors (velocity unit vector e_para and angle unit vector e_perp). In general, it is possible that an attractive interaction may act differently on speed |v| than on the angle phi. I clarify my point by rewriting the equation of motion in polar coordinates in 2D for a total force F:

d|v|/dt = alpha_v * <f_total .="" e_para=“”>

dphi/dt = alpha_phi * <f_total .="" e_perp=“”>

where < . > denotes the scalar product of two vectors.

For a physical force, alpha_v = alpha_phi = 1, but in the general case of a social interaction, these two factors need not be the same. To make things even more complicated, the alphas could depend on relative position angles or even relative orientations. The authors themselves use this approach — for example, in their modeling of the wall force, which they include in Eq. 1 with two separate force terms, one for rotation only (equivalent to setting alpha_v = 0).

Figure 3E clearly shows a negative g_align for |psi| > 120°, with h_ali > 0 for all Delta phi. This indicates that the overall term without sin(Delta phi) is negative. Instead of reducing the heading difference for this particular configuration, it increases Delta phi, which contradicts the statement added in line 248 that it "tends to reduce heading differences." I still do not see how, even with the additional assumption about relative heading difference, this apparent anti-alignment could not be some extra attraction-like response for these particular relative position configurations.

Of course, this is not a simple attractive interaction, as it only acts when the neighbor is behind the focal fish. However, interestingly, the sign change of the alignment interaction in Fig. 3 occurs at psi values where the inferred attraction function has a maximum, and also h_att > h_ali (Fig. 3F). Thus, the apparent slight decrease in g_att for neighbors being behind (|psi| > 120°) might occur because the "anti-alignment" takes over some of the "job" of turning the focal fish toward the neighbor. In the end, what matters is the behavioral response, and it appears to be non-trivially complex. I am now starting to wonder whether the model formulation used in the paper — based on symmetries and empirical observation of anisotropic response, which at first glance makes perfect sense to me — might also constrain inference in a particular way, and whether there could be some alternative general formulation of the interactions that would constrain it differently, thus leading to a potentially different interpretation of the observed anti-alignment pattern.

In the end, I would expect the authors to reconsider their interpretation of anti-alignment, either by providing an actual argument as to why it cannot be an attraction-like response, or providing a short critical discussion on the potential unclear origin, and to correct the imprecise statement in line 248 that it "tends to reduce heading differences."</f_total></f_total>

**********

**Have the authors made all data and (if applicable) computational code underlying the findings in their manuscript fully available?**

The PLOS Data policy requires authors to make all data and code underlying the findings described in their manuscript fully available without restriction, with rare exception (please refer to the Data Availability Statement in the manuscript PDF file). The data and code should be provided as part of the manuscript or its supporting information, or deposited to a public repository. For example, in addition to summary statistics, the data points behind means, medians and variance measures should be available. If there are restrictions on publicly sharing data or code —e.g. participant privacy or use of data from a third party—those must be specified.

Reviewer #1: Yes

Reviewer #2: **No:**  Example computational code used for simulating the model and process experimental results appears not to be made available.

**********

PLOS authors have the option to publish the peer review history of their article (what does this mean?). If published, this will include your full peer review and any attached files.

If you choose “no”, your identity will remain anonymous but your review may still be made public.

**Do you want your identity to be public for this peer review?**  For information about this choice, including consent withdrawal, please see our Privacy Policy.

Reviewer #1: No

Reviewer #2: No

[NOTE: If reviewer comments were submitted as an attachment file, they will be attached to this email and accessible via the submission site. Please log into your account, locate the manuscript record, and check for the action link "View Attachments". If this link does not appear, there are no attachment files.]

**Figure resubmission:**

**Reproducibility:**

To enhance the reproducibility of your results, we recommend that authors of applicable studies deposit laboratory protocols in protocols.io, where a protocol can be assigned its own identifier (DOI) such that it can be cited independently in the future. Additionally, PLOS ONE offers an option to publish peer-reviewed clinical study protocols. Read more information on sharing protocols at https://plos.org/protocols?utm_medium=editorial-email&utm_source=authorletters&utm_campaign=protocols

# Author comment: Author response to Decision Letter 2

**DOI:** 10.1371/journal.pcbi.1014544.r004

23 Jun 2026

Attachment Submitted filename: Response to Reviewers_R2.pdf

# Editor report: Decision Letter 2

**DOI:** 10.1371/journal.pcbi.1014544.r005

**Authors:** Natalia L. Komarova, Nir Gov

8 Jul 2026

Dear Dr. Theraulaz,

We are pleased to inform you that your manuscript 'Closed-loop real-virtual interactions validate 3D model of social coordination in fish' has been provisionally accepted for publication in PLOS Computational Biology.

Before your manuscript can be formally accepted you will need to complete some formatting changes, which you will receive in a follow up email. A member of our team will be in touch with a set of requests.

Please note that your manuscript will not be scheduled for publication until you have made the required changes, so a swift response is appreciated.

IMPORTANT: The editorial review process is now complete. PLOS will only permit corrections to spelling, formatting or significant scientific errors from this point onwards. Requests for major changes, or any which affect the scientific understanding of your work, will cause delays to the publication date of your manuscript.

Should you, your institution's press office or the journal office choose to press release your paper, you will automatically be opted out of early publication. We ask that you notify us now if you or your institution is planning to press release the article. All press must be co-ordinated with PLOS.

Thank you again for supporting Open Access publishing; we are looking forward to publishing your work in PLOS Computational Biology.

Best regards,

Nir Gov

Academic Editor

PLOS Computational Biology

Feng Fu

Section Editor

PLOS Computational Biology

***********************************************************

# Editor report: Acceptance letter

**DOI:** 10.1371/journal.pcbi.1014544.r006

**Authors:** Natalia L. Komarova, Nir Gov

PCOMPBIOL-D-26-00551R2

Closed-loop real-virtual interactions validate 3D model of social coordination in fish

Dear Dr Theraulaz,

I am pleased to inform you that your manuscript has been formally accepted for publication in PLOS Computational Biology. Your manuscript is now with our production department and you will be notified of the publication date in due course.

The corresponding author will soon be receiving a typeset proof for review, to ensure errors have not been introduced during production. Please review the PDF proof of your manuscript carefully, as this is the last chance to correct any errors. Please note that major changes, or those which affect the scientific understanding of the work, will likely cause delays to the publication date of your manuscript.

Soon after your final files are uploaded, unless you have opted out, the early version of your manuscript will be published online. The date of the early version will be your article's publication date. The final article will be published to the same URL, and all versions of the paper will be accessible to readers.

For Research, Software, and Methods articles, you will receive an invoice from PLOS for your publication fee after your manuscript has reached the completed accept phase. If you receive an email requesting payment before acceptance or for any other service, this may be a phishing scheme. Learn how to identify phishing emails and protect your accounts at https://explore.plos.org/phishing.

Thank you again for supporting PLOS Computational Biology and open-access publishing. We are looking forward to publishing your work!

With kind regards,

Kannan R K Kuppusamy, B.TECH BIOTECHNOLOGY

PLOS Computational Biology | Carlyle House, Carlyle Road, Cambridge CB4 3DN | United Kingdom ploscompbiol@plos.org | Phone +44 (0) 1223-442824 | ploscompbiol.org | @PLOSCompBiol
